# Supplementary material for: Evolution of complexity in the zebrafish synapse proteome
Source: Nat Commun. 2017 Mar 2;8:14613. doi: 10.1038/ncomms14613 (PMC5337974; doi:10.1038/ncomms14613)
Supplement: Supplementary Information — Supplementary Figures, Supplementary Table, Supplementary Notes and Supplementary References. [file ncomms14613-s1.pdf]

Supplementary Figures

Supplementary Figure 1

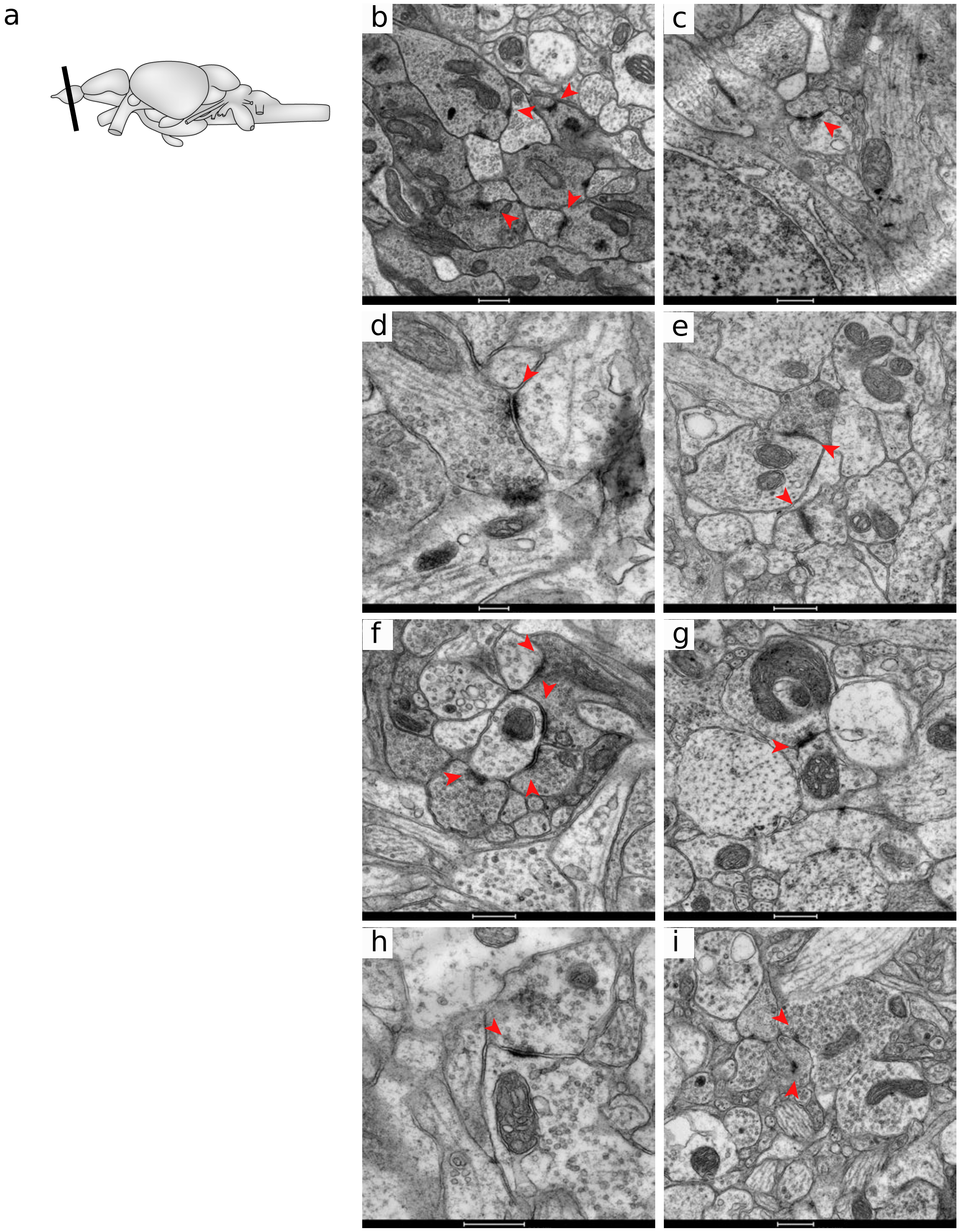

Supplementary Figure 1. Ultrastructure of synapses from Zebrafish olfactory bulb. (a) Schematic representation of the zebrafish brain. The black line indicates the position of the olfactory bulb. (b-i) PSDs found in different areas of the olfactory bulb. Arrowheads indicate PSDs from dendro-dendritic synapses. Scale bars b-c and e-i 500 nm; scale bar d 200 nm.

Supplementary Figure 2

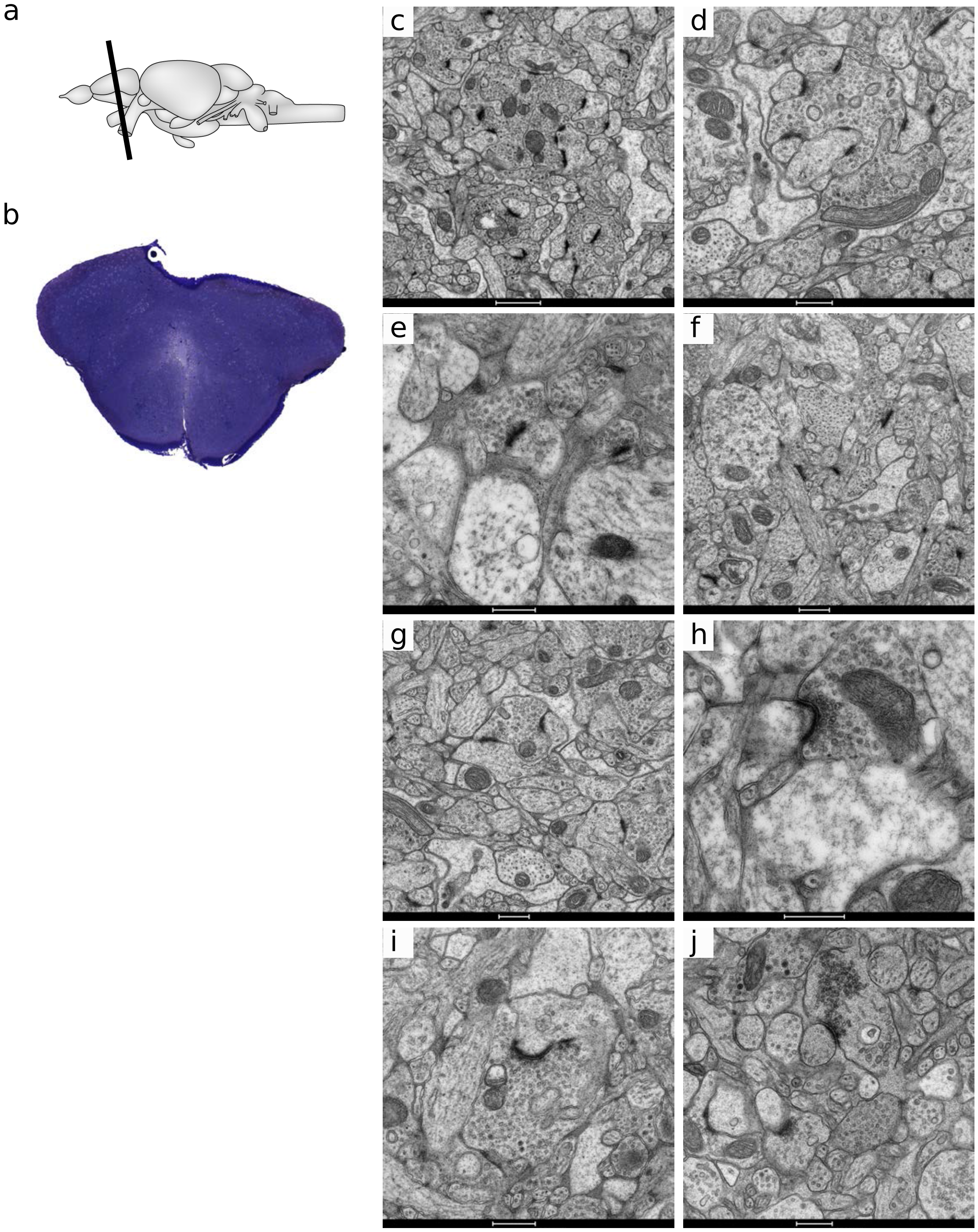

Supplementary Figure 2. Ultrastructure of synapses from Zebrafish telencephalon. (a) Schematic representation of the zebrafish brain. The black line indicates the position of the telencephalon. (b) Coronal semi-thin section of the zebrafish telencephalon stained with toluidine blue. (c-j) PSDs found in different areas of the telencephalon. Scale bar c 1 µm; scale bars d-j 500 nm.

Supplementary Figure 3

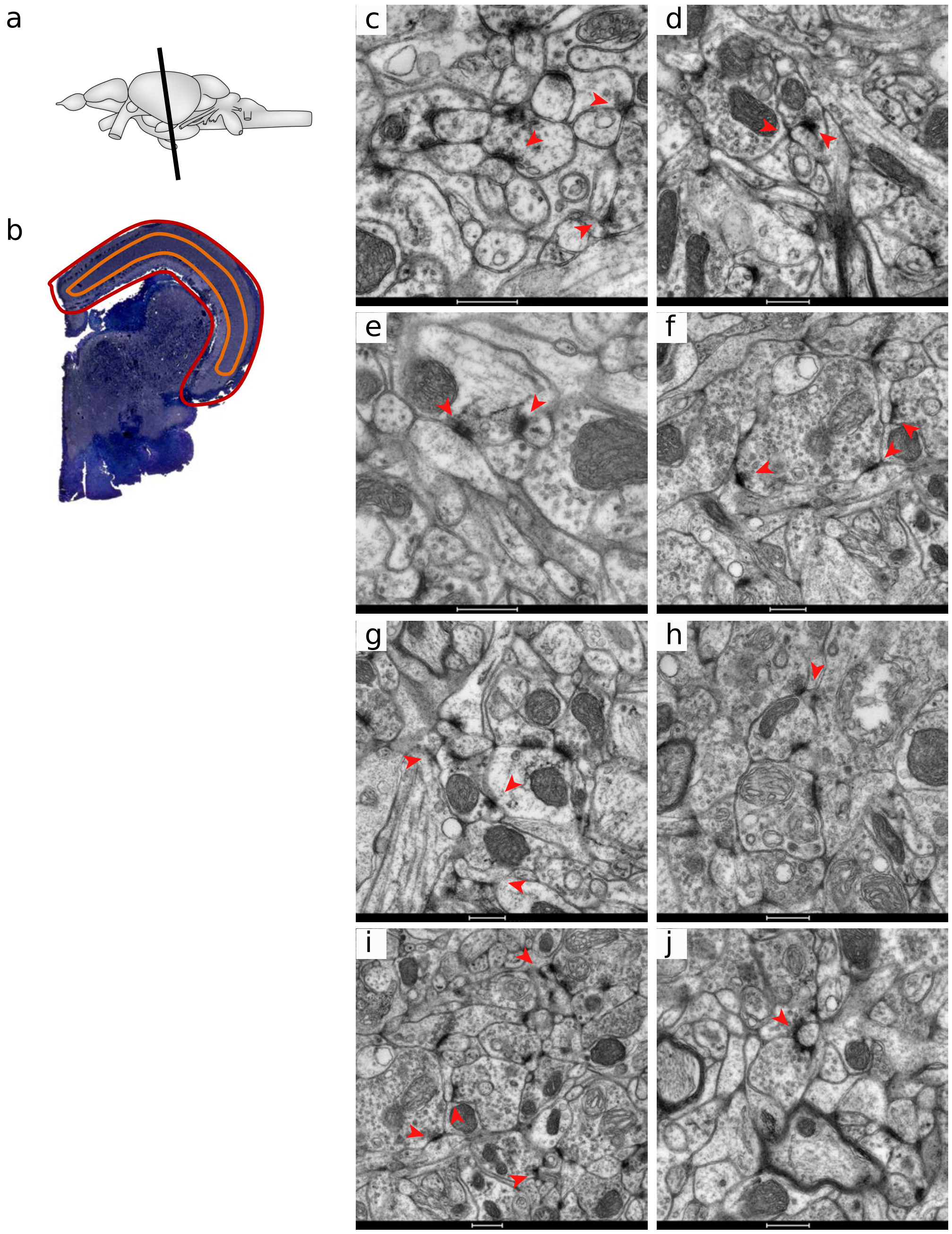

Supplementary Figure 3. Ultrastructure of synapses from Zebrafish optic tectum.

(a) Schematic representation of the zebrafish brain. The black line indicates the position of the midbrain. (b) Coronal semi-thin section of the zebrafish midbrain stained with toluidine blue. The red encircled area corresponds to the optic tectum. The orange line indicates the area where asymmetric synapses were identified. (c-j) PSDs found in different sections of the optic tectum. Arrowheads denote PSDs of postsynaptic elements showing either a small diameter or microtubules underneath. Scale bars c-j 500 nm.

Supplementary Figure 4

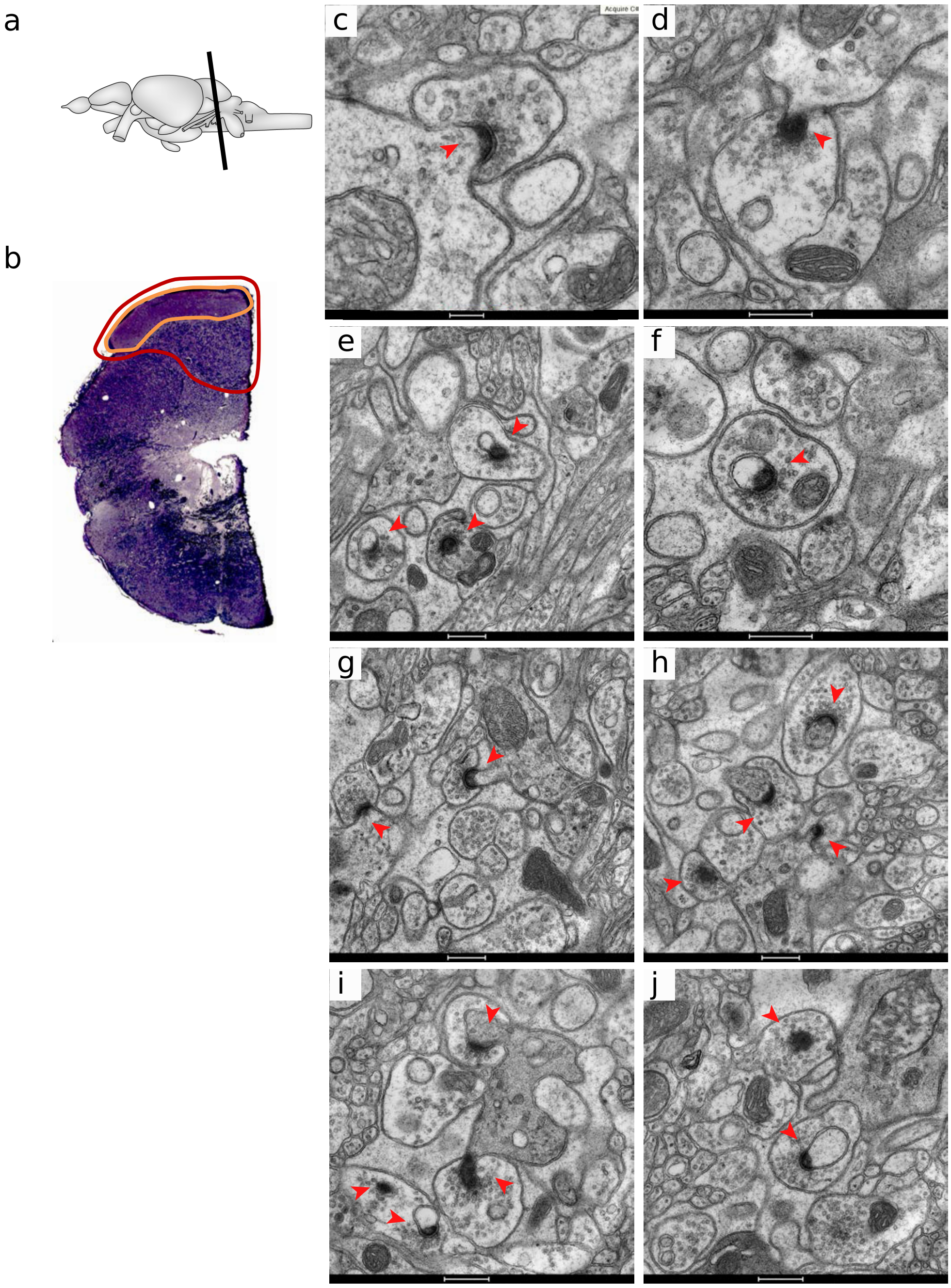

Supplementary Figure 4. Ultrastructure of synapses from Zebrafish cerebellum. (a) Schematic representation of the zebrafish brain. The black line indicates the position of the hindbrain. (b) Coronal semi-thin section of the zebrafish hindbrain stained with toluidine blue. The red encircled area corresponds to the cerebellar corpus. The orange line indicates the area where asymmetric synapses were identified. (c-j) PSDs found in different sections of the cerebellum. Arrowheads indicate PSDs showing the curved morphology with the presynaptic element surrounding the postsynaptic spine. Scale bar c 200 nm; scale bars d-j 500 nm.

Supplementary Figure 5

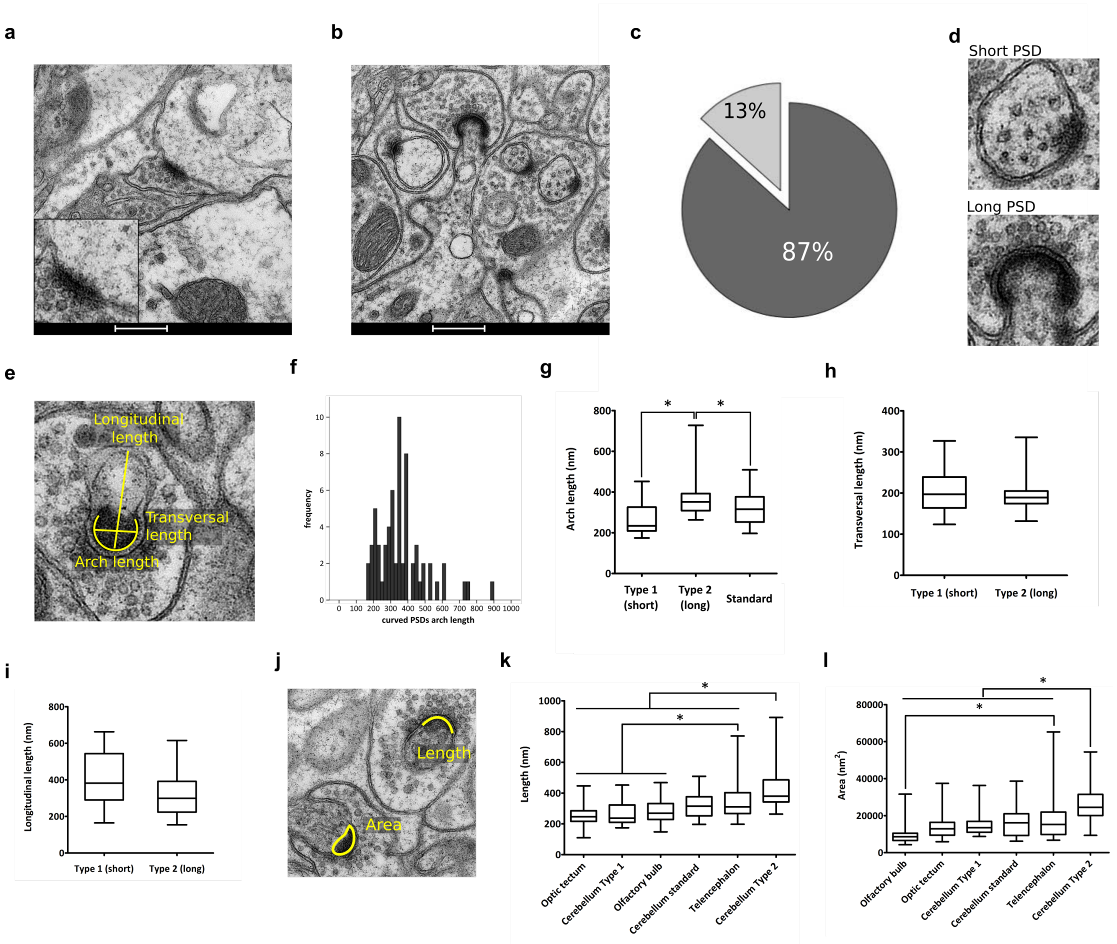

Supplementary Figure 5. Morphological characteristics of zebrafish PSDs

a. Synapses from the cerebellar corpus with morphological characteristics similar to those observed in mammals and other zebrafish brain regions, including flat PSDs. Scale Bar 500nm.

b. Synapses from the cerebellar corpus with presynaptic elements engulfing the postsynaptic terminal and highly curved PSDs. Scale Bar 500nm.

c. Proportion of flat (light grey) and curved (dark grey) PSDs found at the cerebellar corpus of zebrafish brain.

d. Representative images of the two types of PSD found amongst curved ones: type 1 (top image) and type 2 (bottom image).

e. Representation of the three measures taken on cerebellar synapses: PSD arch length, PSD transversal length and spine longitudinal length.

f. Histogram showing distribution of arch length for curved PSDs.

g. PSD arch length differences between the three types of PSDs found at the cerebellar corpus ($p < 0.05$, Kruskal-Wallis test). Type 1 n = 24 PSDs; type 2 n = 40 PSDs; standard n = 10 PSDs.

h. PSD transversal length differences between the two types of curved PSDs found at the cerebellar corpus. Type 1 n = 24 PSDs; type 2 n = 40 PSD.

i. Spine longitudinal length differences between the two types of curved PSDs found at the cerebellar corpus. Type 1 n = 24 spines; type 2 n = 40 spines.

j. Representation of the two measures taken on PSD from all brain regions: length and area.

k. Differences in PSD length between brain regions ($p < 0.05$, Kruskal-Wallis test). Optic tectum n = 55 PSDs; Cerebellum type 1 n = 25 PSDs; Olfactory bulb n = 52 PSDs; Cerebellum standard n = 10 PSDs; Telencephalon n = 86 PSDs; Cerebellum type 2 n = 40 PSDs.

l. Differences in PSD area between brain regions ($p < 0.05$, Kruskal-Wallis test). Olfactory bulb n = 53 PSDs; Optic tectum n = 53 PSDs; Cerebellum type 1 n = 26 PSDs; Cerebellum standard n = 10 PSDs; Telencephalon n = 87 PSDs; Cerebellum type 2 n = 37 PSDs.

Supplementary Table 1

	Total N*	Median	Max	Min
**Area (nm^2)**				
olfactory bulb	53	8590.0	31616.7	4284.9
telencephalon	87	15258.3	65249.2	6745.7
optic tectum	53	12876.9	37449.3	5913.3
cerebellum standard	10	16155.2	38637.1	6156.9
cerebellum type 1 (short)	26	13517.5	36301.8	8740.0
cerebellum type 2 (long)	37	24514.8	54409.1	9327.3
**Length (nm)**				
olfactory bulb	52	269.4	468.1	148.2
telencephalon	86	310.4	771.0	197.6
optic tectum	55	246.7	447.2	110.2
cerebellum standard	10	315.1	509.0	196.8
cerebellum type 1 (short)	25	237.0	452.0	174.5
cerebellum type 2 (long)	40	380.3	891.7	263.6
**Arch length (nm)**				
cerebellum standard	10	315.1	509.0	196.8
cerebellum type 1 (short)	24	234.2	452.0	174.5
cerebellum type 2 (long)	40	380.3	891.7	263.6
**Transversal length (nm)**				
cerebellum type 1 (short)	24	49.5	197.0	326.8
cerebellum type 2 (long)	40	39.1	191.4	335.3
**Longitudinal length (nm)**				
cerebellum type 1 (short)	24	382.0	662.8	164.6
cerebellum type 2 (long)	40	335.5	615.5	154.6

*N = number of analysed PSDs

Supplementary Table 1. PSD morphological metrics.

Supplementary Figure 6

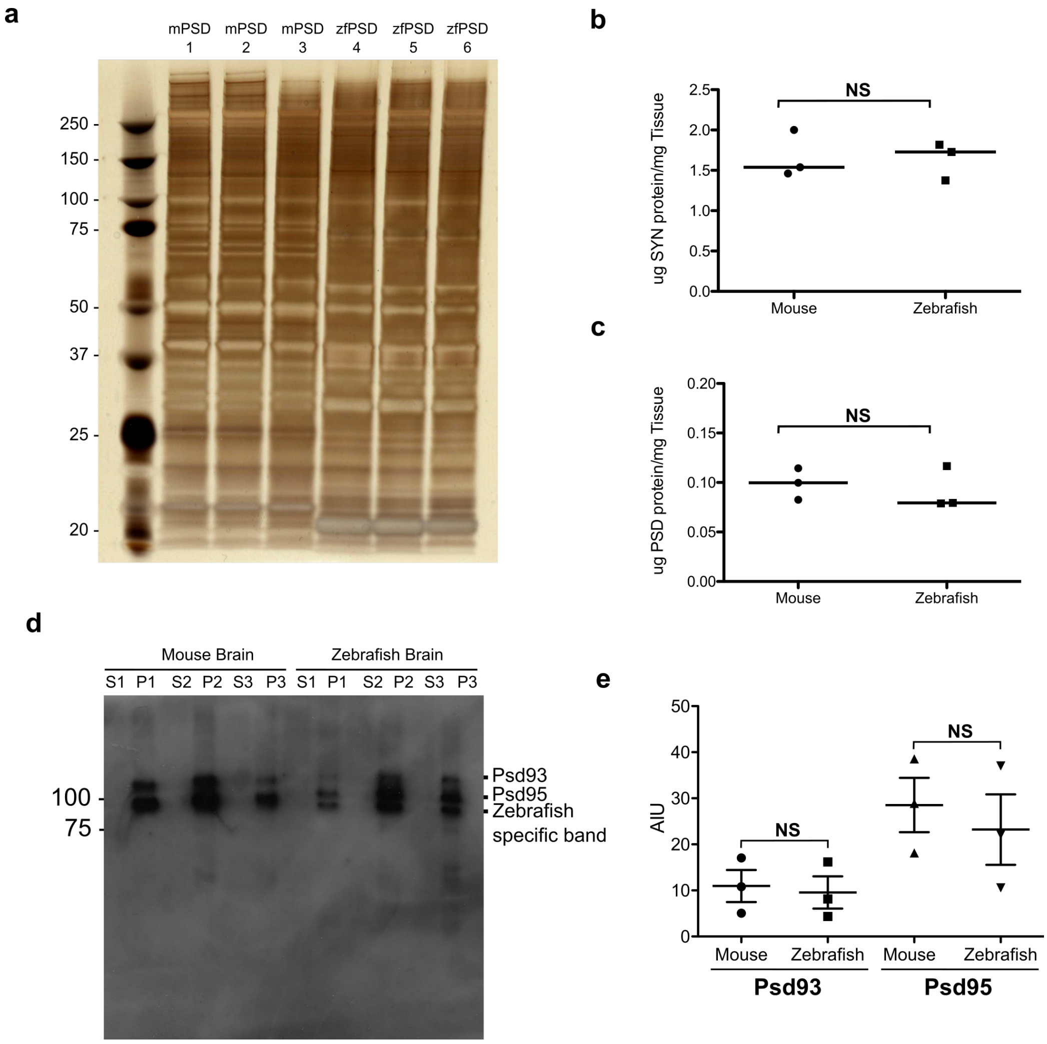

Supplementary Figure 6. Isolation of postsynaptic densities from mouse and zebrafish brains.

a. SDS-PAGE electrophoresis of the three PSD fractions isolated from mouse brain (lanes 1-3) and zebrafish brain (lanes 4-6).

b. Protein yield for synaptosomal (SYN) preparations from mouse and zebrafish brains.

c. Protein yield for postsynaptic density (PSD) preparations from mouse and zebrafish brains.

d. Immunoblot of synaptosomal (S) and PSD (P) fractions for PSD95 protein in triplicate samples from mouse and zebrafish brains. Representative image of 3 western blots.

e. Quantification of western blot image in (d) of bands corresponding to Psd93 and Psd95 in postsynaptic density fractions from both species (AIU: arbitrary units of intensity).

Supplementary Figure 7

a

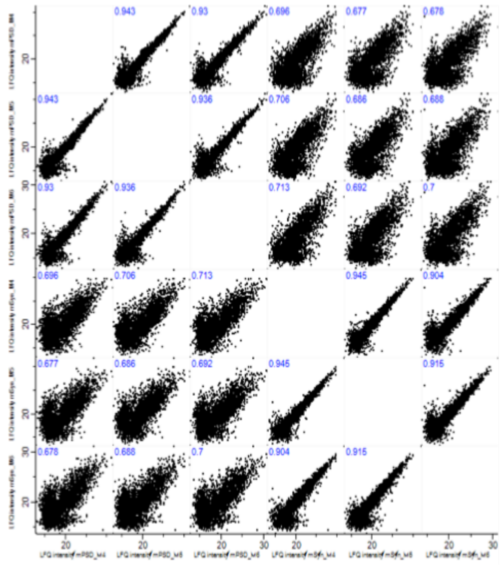

b

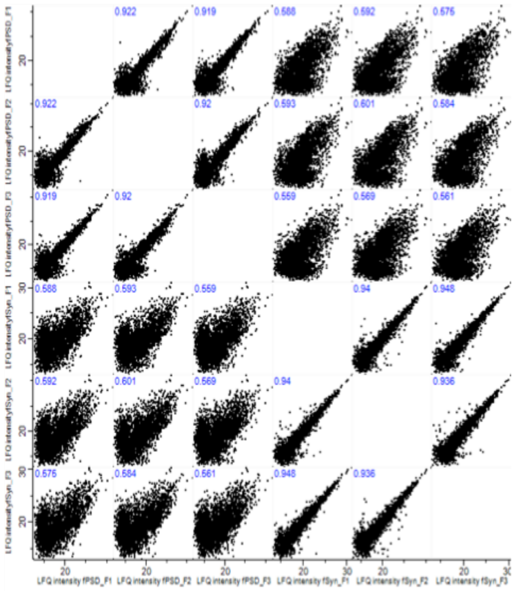

Supplementary Figure 7. Label Free Quantification (LFQ) intensities of zebrafish and mouse synaptic proteins.

a. Multiscatter plot of protein LFQ intensities calculated from data obtained from proteomic analysis of Zebrafish synaptosome and PSD samples. Pearson correlation coefficients are shown for each comparison and were >0.90 for biological replicates in each group.

b. Multiscatter plot of protein LFQ intensities calculated from data obtained from proteomic analysis of mouse synaptosome and PSD samples. Pearson correlation coefficients are shown for each comparison and were >0.90 for biological replicates in each group.

Supplementary Figure 8

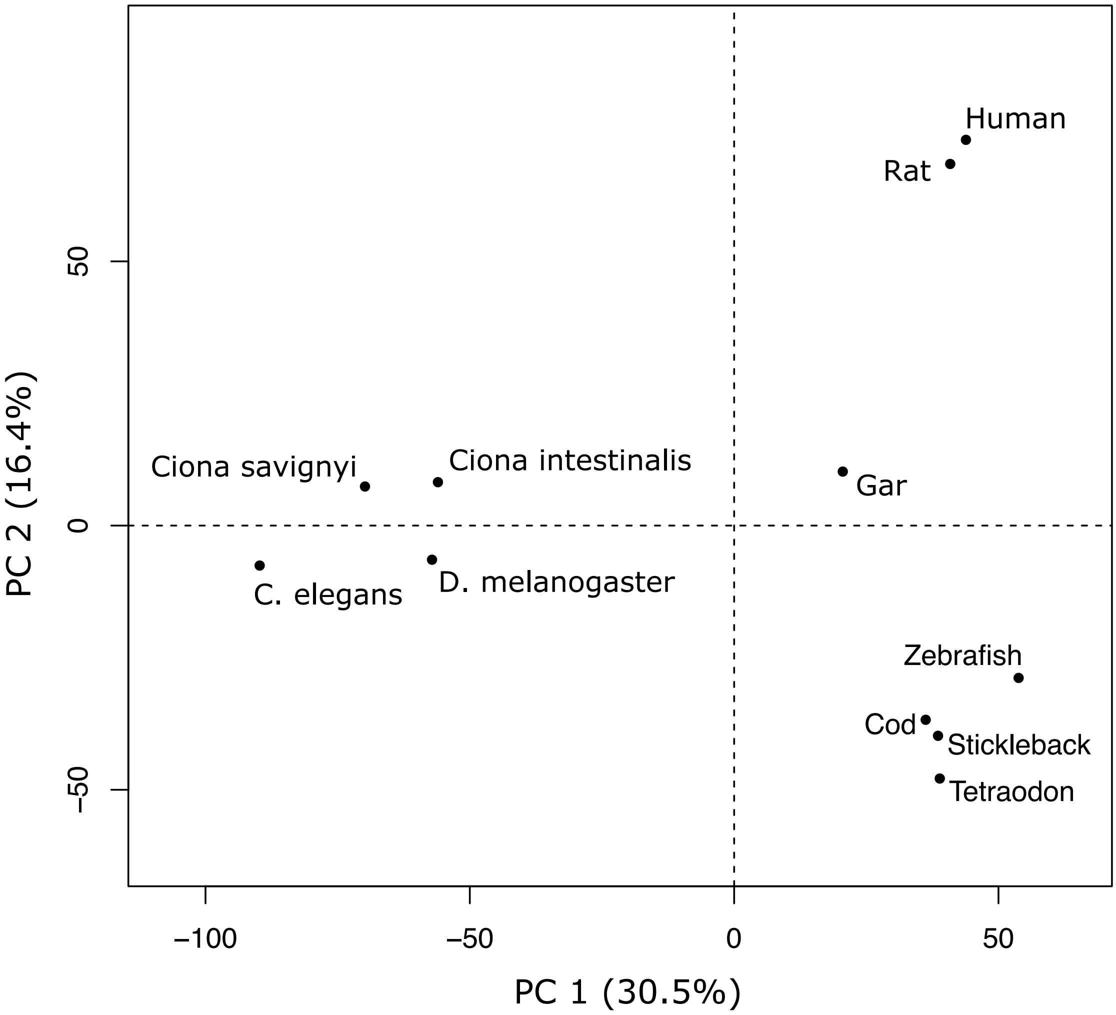

Supplementary Figure 8. Principal components analysis (PCA) of the ratio of homologs.

Biplot of the first two components of a PCA model comparing ratios of homolog counts in gene families for multiple species including additional fish.

Whole genome data was obtained from Ensembl and the number of homologs between mouse and each species for gene families were determined. The resulting matrix of homolog ratios for all species were compared using PCA.

The invertebrate and chordate species shows clear clustering away from the vertebrate species. This analysis supports the assumption that gene family size is dominated by vertebrate lineage whole genome duplication events. The separation of the Spotted Gar (*Lepisosteus oculatus*) whose lineage diverged before the additional teleost specific WGD[6] places it in the same quadrant as the mammalian species supporting the premise that the major event in genome evolution of the teleost fish was the additional WGD event.

Supplementary Figure 9

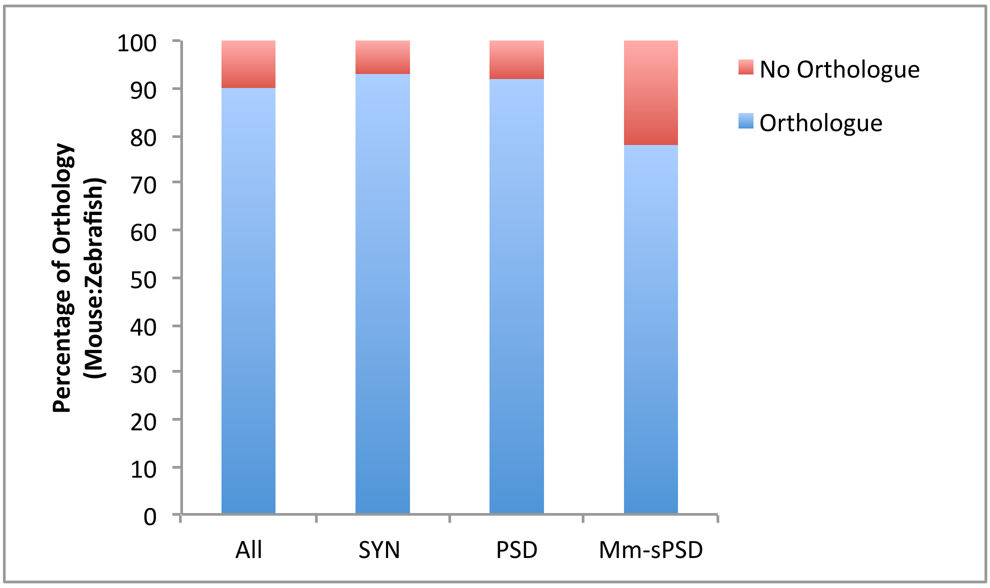

Supplementary Figure 9. Stacked column chart for orthologue percentage of mouse synaptic proteins in zebrafish genome.

Section of column in blue represents the percentage of mouse proteins with an orthologue in the zebrafish genome. The section in red represents the percentage of mouse proteins without an orthologue in the zebrafish genome. X-axis legends correspond to. All proteins identified in mouse (All), proteins found in synaptosomes (SYN), proteins found in PSDs (PSD) and protein from mouse PSD absent from zebrafish PSD or synaptosomes (Mm-sPSD).

Supplementary Figure 10

a. Vesicle fusion with membrane

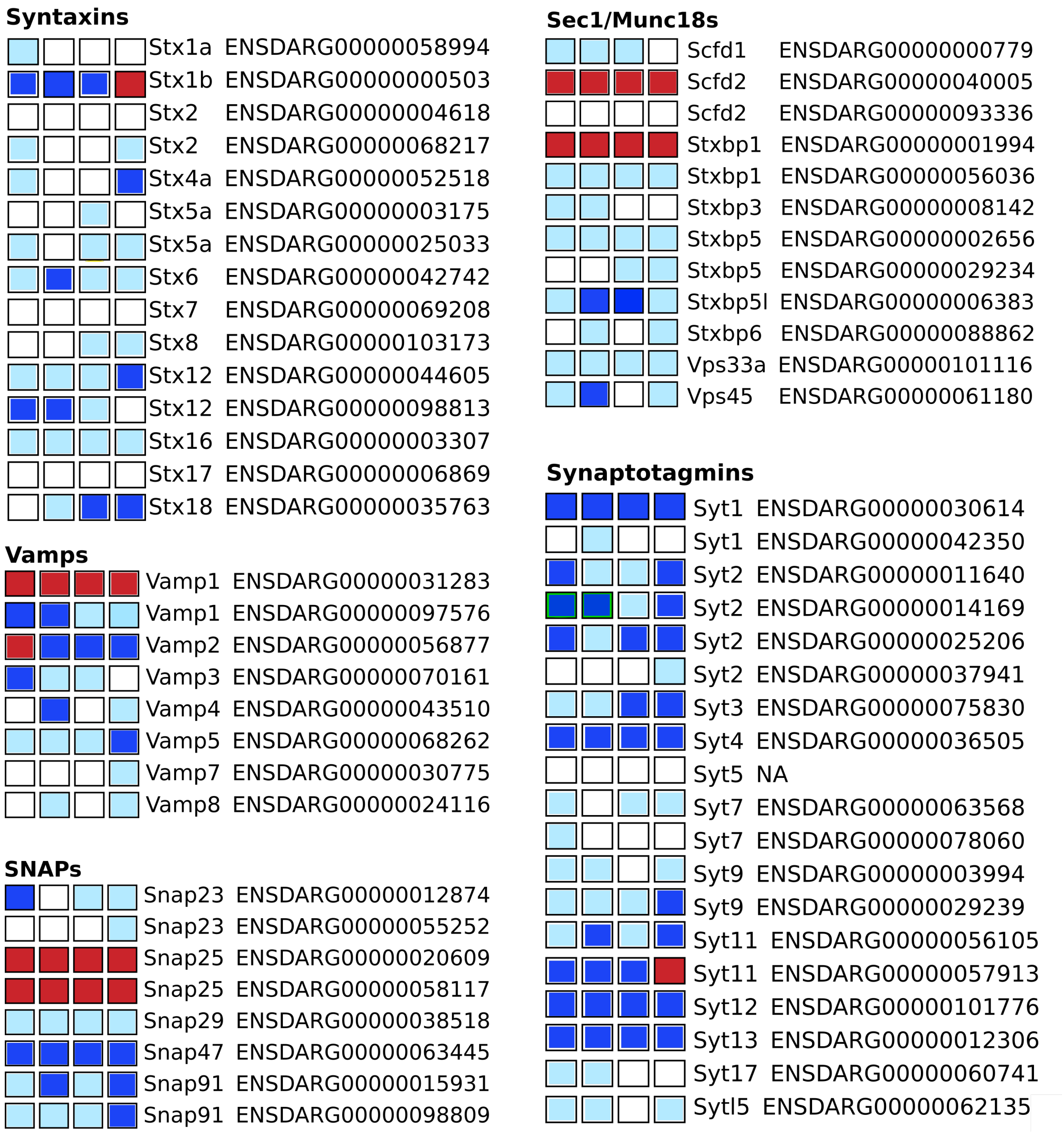

b. Membrane tethering & Membrane budding and scission.

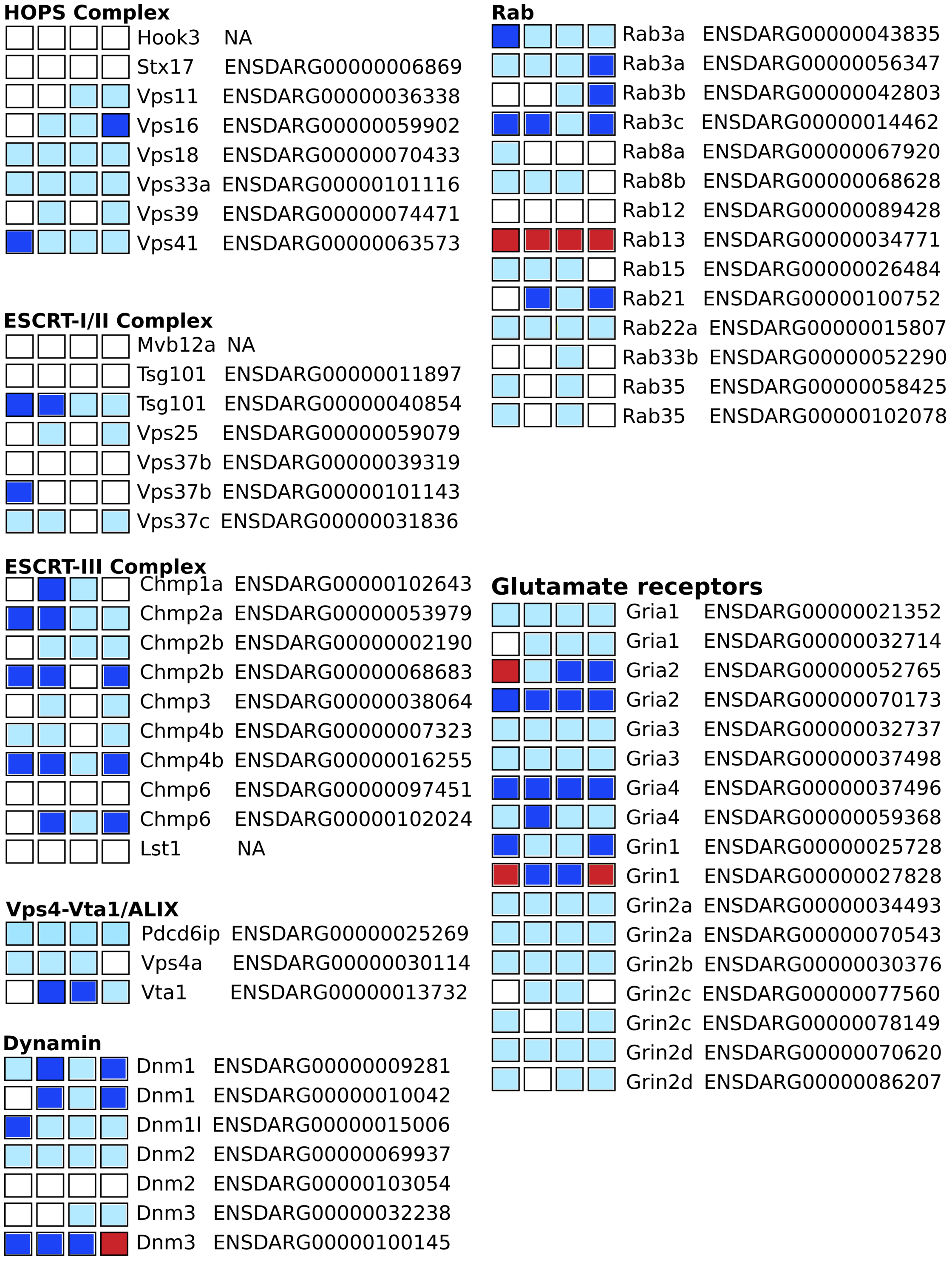

Supplementary Figure 10. Zebrafish whole brain mRNA levels corresponding to proteins involved in SNARE, HOPS, ESCRT complexes and related proteins. mRNA expression as measured by transcripts per million (TPM). For each gene four biological replicate RNASeq experiments are shown as columns. Colour code

reflects mRNA abundance: Red, >50 TPM; Dark blue, between 10 and 50 TPM, Light blue between 1 and 10 TPM and White, less than 1 TPM.

The following software was used to process RNAseq data: adapters were removed from the raw reads using Cutadapt[7]. TopHat2[8] was used as a wrapper for the alignment programme Bowtie2[9] to map sequence reads to the reference genome (Danio_rerio.GRCz10.86 obtained via Ensembl), reads were converted into counts using HTSeq[10], and converted to transcript per million (TPM)[11]. For comparison of each gene, the average expression (mean transcripts per million (TPM) from four whole brain biological replicates) was determined for each gene. Where multiple transcripts for a given gene are known these were combined to result in a single mean TPM per gene.

a. Expression of genes encoding proteins associated with Vesicle fusion with membrane

b. Expression of genes encoding proteins associated with Membrane tethering & Membrane budding and scission**.** Genes encoding glutamate receptors are shown as a comparison.

Supplementary Figure 11

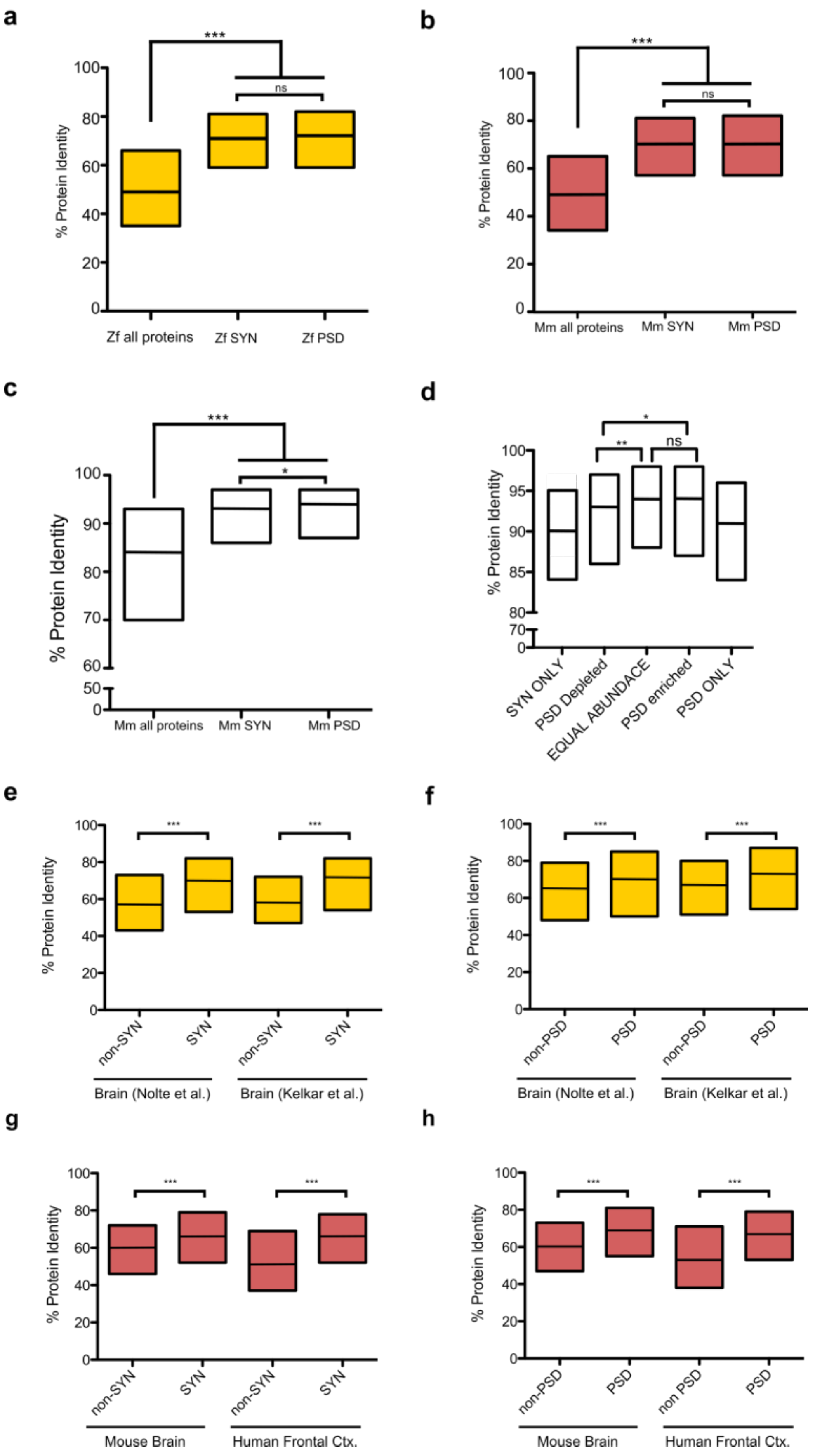

Supplementary Figure 11. Conservation of SYN and PSD proteins along vertebrate evolution.

a. Box plots for the percentage of protein identity since the last common ancestor between zebrafish and mouse in the following zebrafish protein groups: all proteins encoded in the genome, brain synaptosomes (Zf-SYN) and brain postsynaptic densities (Zf-PSD).

b. Box plots for the percentage of protein identity since the last common ancestor between zebrafish and mouse in the following mouse protein groups: all proteins encoded in the genome, brain synaptosomes (Mm-SYN) and brain postsynaptic densities (Mm-PSD).

c. Box plots for the percentage of protein identity since the last common ancestor between mouse and human in the following protein groups: all mouse proteins encoded in the genome, mouse synaptosomal (Mm-SYN) and postsynaptic density (Mm-PSD) proteins.

d. Box plots for the percentage of protein identity since the last common ancestor between mouse and human of proteins found only in the mouse synaptosomal fraction, with equal abundance in both fractions or depleted, enriched or only found at the PSD**.**

e. Box plots for the percentage of protein identity since the last common ancestor between zebrafish and mouse of proteins identified in the zebrafish brain divided into those found (SYN) or absent (non-SYN) in our synaptosomal preparation. Data from two independent zebrafish brain proteomes are used[1-3].

f. Box plots for the percentage of protein identity since the last common ancestor between zebrafish and mouse of proteins identified in the zebrafish brain divided into those found (PSD) or absent (non-PSD) in our postsynaptic density preparation. Data from two independent zebrafish brain proteomes are used[2,3].

g. Box plots for the percentage of protein identity since the last common ancestor between zebrafish and mouse of proteins identified in a mouse brain proteome[4] and a human frontal cortex proteome[1] divided into those found (SYN) or absent (non-SYN) in our synaptosomal preparation.

h. Box plots for the percentage of protein identity since the last common ancestor between zebrafish and mouse of proteins identified in a mouse brain proteome[4] and a human frontal cortex proteome[1] divided into those found (PSD) or absent (non-PSD) in our postsynaptic density preparation.

All distribution compared using the Mann-Whitney U test (*** $p < 0.001$; ** $p < 0.01$ and * $p < 0.05$).

Supplementary Note 1. Zebrafish synapse ultrastructure

Transmission electron microscopy images of zebrafish brain suggest differential morphological features of postsynaptic densities (PSD) between zebrafish brain regions, including olfactory bulb, telencephalon, optic tectum and cerebellum (Supplementary Fig. 1-5). The most noticeable changes were found in the cerebellar corpus (Supplementary Fig. 4) where two well-differentiated types of PSDs could be observed. Flat PSDs, looking similar to mammalian ones and to those found in other zebrafish brain regions (standard, Supplementary Fig. 5a), and curved PSDs, presenting a presynaptic bouton greatly surrounding the postsynaptic element (Supplementary Fig. 5b). Curved PSDs were prominent in the cerebellum, accounting for 87% of the total (Supplementary Fig. 5c). Moreover, two additional subsets of curved PSDs could be observed: short (Type 1) and long (Type 2) (Supplementary Fig. 5d). To provide empirical evidence that these PSDs were indeed different in their morphology, we measured several parameters regarding their shape and size (Supplementary Fig. 5 and Supplementary Table 1). As we expected, the measured PSD lengths (arch length, see Supplementary Fig. 5e) showed a bimodal distribution (Supplementary Fig. 5f), indicating the presence of two subpopulations. In this distribution type 2 curved PSDs were significantly longer than type 1 ($p < 0.05$, Supplementary Fig. 5g and Supplementary Table 1). Actually, long PSDs were also significantly longer than flat (standard) cerebellum PSDs ($p < 0.05$, Supplementary Fig. 5g and Supplementary Table 1). Additionally, to prove that the observed size differences were not due to the depth of the tissue section, we measured the transversal and the longitudinal lengths of postsynaptic spines containing curved PSDs (Supplementary Fig. 5e). Indeed, no differences were found for longitudinal or transversal lengths between postsynaptic spines of type 1 and type 2 curved PSDs (Supplementary Fig. 5h-i and Supplementary Table 1), indicating they represent two different populations of curved PSDs.

Finally, since the previous analysis provided valuable information on PSD morphology, we performed similar measurements on the other zebrafish brain regions. In this case we measured two variables: PSD length and area (Supplementary Fig. 5j and Supplementary Table 1). This showed that the largest forebrain PSDs were found in telencephalon synapses ($p < 0.05$, Supplementary Fig. 5k-l). Moreover, PSDs from the olfactory bulb, optic tectum, cerebellum type 1 and cerebellum standard were similar in size. Finally, type 2 curved PSDs from the cerebellar corpus were significantly the largest ones of the whole zebrafish brain ($p < 0.05$, Supplementary Fig. 5k-l).

Supplementary Note 2. Proteins identified by mass spectrometry and filtering criteria used to define synaptosomal and PSD datasets

The exact same criteria were applied to mouse and zebrafish mass spectrometry data. In the first place a protein FDR of 0.01 and peptide FDR of 0.01 were used as protein identification level cut offs. This generated a first list of proteins that included a number of duplicated entries due to the identification of different isoforms for 134 and 151 mouse and zebrafish proteins. After removal of these duplicated entries we generated a first list of 4162 (Supplementary Data 1, first sheet) and 4486 (Supplementary Data 2, first sheet) unique proteins found in mouse and zebrafish respectively. To establish the final list of SYN and PSD proteins we decided to only consider proteins that were found with at least one unique peptide in each of the three SYN or PSD replicas. This filtering criterion was used to minimise the presence of biochemical contaminants in the final datasets, as these are less likely to appear in triplicate. This cut-off criterion reduced the number of proteins finally accepted as mouse and zebrafish synaptic proteins to 3579 and 3840 respectively.

Finally, using label free quantification data we established which proteins were enriched or depleted in the PSD relative to the synaptosomal fraction. Proteins significantly depleted from the PSD were not considered for the final list of PSD proteins, as these are also more likely to be contaminants due to the biochemical fractionation process.

All these criteria were used to define the final list of components of in mouse and zebrafish synaptosomal (SYN) and PSD fractions. Overall we document 3223 and 2128 proteins in mouse SYN and PSD fractions (Supplementary Data 1, third sheet) and 3640 and 1758 proteins (Supplementary Data 2, third sheet) in the corresponding structures from zebrafish (Figure 2).

Supplementary Note 3. Evolutionary origins of species differences in SYN and PSD proteomes

We have shown that zebrafish presents larger families than mouse (Fig. 3a,b). This has led us to hypothesize that the teleost-specific whole genome duplication (TGD) has been the major force driving this increase in family size. Nevertheless, these could also be consequence of gene loss in mouse or, alternatively, due to tandem duplications occurred in the fish lineages leading to zebrafish after the TGD.

We have also shown that mammalian PSD proteins absent from zebrafish include protein types with important synaptic functions. Here we hypothesized that after fish

diverged from the rest of vertebrates the PSD proteome has incorporated new proteins. Nevertheless, loss of the genes coding for these proteins in the zebrafish genome is an alternative explanation.

To clarify these points we have performed two analyses:

i) Evaluate family size in other fish species appeared after the TGD.
ii) Identify mouse synaptic proteins without an orthologue in the zebrafish genome as a measure of gene loss in zebrafish.

i) In order to consider family size in bony fish species appeared after the TGD we have looked for the number of orthologs per gene family between mouse and three fish species (Cod, *Gadus morhua*; Tetraodon, *Tetraodon nigroviridis* and Stickleback, *Gasterosteus aculeatus*) beyond zebrafish. In parallel we have done the same analysis on a bony fish whose lineage appeared before the TGD (Spotted Gar, *Lepisosteus oculatus*), in two vertebrate species (*Homo sapiens* and *Rattus norvegicus*), two chordates (*Ciona intestinalis* and *Ciona savignyi*) and in two invertebrate species (*Drosophila melanogaster* and *Caenorhabditis elegans*). We have found that the 2:1 ratio (Spp:Mouse) in orthologue count of SYN and PSD proteins is increased in all fish appeared after the TGD but not in the Spotted Gar. When doing a principal components analysis (PCA) and plotting the first two components, the fish species appearing after the TGD cluster together while the Spotted Gar clusters with the vertebrate (Supplementary Fig. 8). This data clearly indicates that gene families of proteins expressed at the synapse tend to be increased in all fish species appearing after the TGD. Supporting the idea that the TGD has shaped synaptic gene family size.

ii) We have looked for zebrafish orthologs of mouse synaptic proteins to evaluate gene loss in zebrafish. Importantly most mouse synaptic proteins have an orthologue identified in the zebrafish genome, thus discarding gene loss as the main explanation for the differences observed between species. For instance, if all mouse proteins are considered 90% have an orthologue in the zebrafish genome or of mouse PSD specific proteins (Mm-sPSD) 80% also show an orthologue in zebrafish (Supplementary Fig. 9).

Overall these findings indicate that the teleost-specific whole genome duplication is most likely the major driving force behind the expansion of gene families for proteins

expressed at the zebrafish synapse. Also, that major gene loss has not occurred between mouse and zebrafish, at least for genes coding for proteins expressed at the synapse. Thus differences in proteome composition between species is not mainly consequence of gene loss.

Supplementary References

1. Kim, M.-S. *et al.* A draft map of the human proteome. *Nature* **509,** 575–581 (2014).
2. Nolte, H. *et al.* Global protein expression profiling of zebrafish organs based on in vivo incorporation of stable isotopes. *J Proteome Res* **13,** 2162–2174 (2014).
3. Kelkar, D. S. *et al.* Annotation of the zebrafish genome through an integrated transcriptomic and proteomic analysis. *Mol Cell Proteomics* **13,** 3184–3198 (2014).
4. Wang, Y. *et al.* A molecular brake controls the magnitude of long-term potentiation. *Nat Comms* **5,** 3051 (2014).
5. Durinck, S., Spellman, P. T., Birney, E. & Huber, W. Mapping identifiers for the integration of genomic datasets with the R/Bioconductor package biomaRt. *Nat Protoc* **4,** 1184–1191 (2009).
6. Braasch, I. *et al.* The spotted gar genome illuminates vertebrate evolution and facilitates human-teleost comparisons. *Nat Genet* **48,** 427–437 (2016).
7. Martin, M. Cutadapt removes adapter sequences from high-throughput sequencing reads. *EMBnet.journal;* **17**, No 1. DOI: http://dx.doi.org/10.14806/ej.17.1.200 (2011).
8. Kim D, Pertea G, Trapnell C, Pimentel H, Kelley R, Salzberg SL. TopHat2: accurate alignment of transcriptomes in the presence of insertions, deletions and gene fusions. Genome Biology, **14**, R36 (2013).
9. Langmead B, Salzberg S. Fast gapped-read alignment with Bowtie 2. Nature Methods. **9**, 357-359 (2012).
10. Simon Anders, Paul Theodor Pyl, Wolfgang Huber; HTSeq—a Python framework to work with high-throughput sequencing data. *Bioinformatics*, **15**, 166-169 (2015).
11. Wagner, G.P., Kin, K. & Lynch, V.J. Measurement of mRNA abundance using RNA-seq data: RPKM measure is inconsistent among samples. *Theory Biosci*. **131**, 281-285 (2012).
